# A prospective randomized controlled trial assessing the effect of music on patients’ anxiety in venous catheter placement procedures

**DOI:** 10.1038/s41598-022-10862-0

**Published:** 2022-04-28

**Authors:** Florian Nima Fleckenstein, Agnes Klara Böhm, Federico Collettini, Anne Frisch, Willie Magnus Lüdemann, Elif Can, Bernhard Gebauer, Martin Jonczyk

**Affiliations:** 1 Department of Diagnostic and Interventional Radiology, Charité-Universitätsmedizin Berlin, Freie Universität Berlin and Humboldt-Universität Zu Berlin, Augustenburger Platz 1, 13353 Berlin, Germany; 2 Berlin Institute of Health at Charité-Universitätsmedizin Berlin, BIH Biomedical Innovation Academy, BIH Charité Clinician Scientist Program, Berlin, Germany

**Keywords:** Randomized controlled trials, Quality of life, Palliative care, Pain management, Palliative care

## Abstract

The aim of the study was to assess the influence of music on anxiety levels compared to standard patient care in patients undergoing venous catheter placement procedures. This prospective randomized controlled trial included patients undergoing placement procedures for peripherally inserted central venous catheters (PICC), ports and central venous catheters (CVC). Patients were randomly assigned to a music intervention group (MIG) and a control group (CTRL). State and trait anxiety levels were assessed as primary outcome using the state-trait anxiety inventory (STAI) before and after the procedures. Secondary outcomes comprised averaged heart rate for all participants and time of radiological surveillance for port placement procedures exclusively. 72 participants were included into the final analysis (MIG *n* = 40; CTRL *n* = 32). All procedures were successful and no major complications were reported. Mean levels for post-interventional anxieties were significantly lower in the MIG compared to the CTRL (34.9 ± 8.9 vs. 44 ± 12.1; *p* < 0.001). Mean heart rate in the MIG was significantly lower than in the CTRL (76.1 ± 13.7 vs. 93 ± 8.9; *p* < 0.001). Procedure time for port implantation was significantly longer in the MIG by 3 min 45 s (*p* = 0.031). Music exposure during central venous catheter placement procedures highly significantly reduces anxiety and stress levels and can be used to improve patients’ overall experience in the angio suite.

## Introduction

Pain and anxiety management in the field of interventional radiology (IR) is especially challenging since most patients are usually treated while being full conscious. Peri- and post-procedural anxiety is therefore a frequent problem in these procedures^1–3^.

In general, high anxiety levels are associated with decreased pain tolerance, increased use of analgesics as well as sedation, hampering post-therapeutic outcomes by lengthening recovery and hospital discharge time^4,5^.

Music is an integral part of human life and the perception of its elements comprising melody, sound, harmony, rhythm, texture, structure and expression is unique for humankind^6,7^. The exposure to a welcomed auditory stimulus impacts the expression of hormones, such as cortisol, and effects the autonomic nervous system by decreasing sympathetic while simultaneously increasing parasympathetic nerve activity^8,9^. Yet, it’s influence expands beyond a physical level, comprising pain intensity and stress response, to an emotional level. In the context of peri-procedural outcomes, there are three separate effects currently being accounted for: emotional comfort, distraction from pain as well as an ability to counteract feelings of subjection and loss of control by acting as a familiar stimulus in the foreign hospital environment^5,10^. Consecutively, music might alleviate anxiety, while anxiety in itself is known to affect the pain experience^5,11^. In the context of medical procedures, this interaction can lead to a decreased need for sedation and analgesics^11–14^.

Current knowledge about the general influence of music on analgesia derives from neuroimaging studies, according to which lowered pain sensitivity is associated with altered endogenous opioid levels^6,11,15^.

Music interventions are of interest as they embody a widely applicable, easily available, uncomplicated-to-use, inexpensive, non-invasive, non-interacting and safe environmental adjustment to optimize patient care^8,11^. Yet, despite being already used for individuals of all ages and in various specialties, such as cancer care, pain management, dementia and palliative care, music interventions have not been integrated into the peri-interventional routine of IR on a scientific basis^4,9^.

The aim of this study is to evaluate the effects of music on anxiety levels of patients receiving a venous catheter placement procedure in order to maximize the quality of patient care.

## Methods and materials

### Design and randomization

This prospective randomized controlled study was conducted in the radiological department of *blinded* from November 2019 to June 2020. A total of 176 patients were approached for meeting eligibility. The study complies with the Declaration of Helsinki and was approved by the ethics committee of the Charité–Universitätsmedizin Berlin (no. EA4/079/16) on 06/29/2016. The trial was further registered with the German Clinical Trials Register (no. DRKS00026003 on 17/08/2021 accessible under https://www.drks.de/). All patients were informed and gave written consent to participate in the study. Randomization was achieved by the use of a computer-generated randomization programme (https://www.randomizer.org/).

### State and trait anxiety inventory (STAI)

The STAI is a standardized, self-reported psychometric test with high levels of reliability and validity^4,15–17^. The three questionnaires consist of 20 items each, which ought to be rated on a four-point Likert scale. A score of 40 or higher on the scale from 20 to 80 is considered as high-level of anxiety. Whereas the pre- and post-interventional state anxiety inventories (SAI) deal with acute levels of anxiety before and after the evaluated interventions, a third one, the trait anxiety inventory (TAI), records the overall extent of anxiety as a character trait prior to the intervention. The German version of the STAI has a Cronbach’s α between 0.90 and 0.94 for the state scale and between 0.88 and 0.94 for the trait scale^17^.

### Procedures

Subject of this study are patients undergoing three different IR procedures: central venous catheter (CVC), peripherally inserted central catheter (PICC) and port catheter (port) placement procedures. All three devices enable stable, mid to long-term venous access that is required for complex and multimodal intravenous therapy, especially for oncological patients^18^.

### Inclusion and exclusion criteria

Inclusion criteria were age of majority, hemodynamic stability, German speaking and ability to consent participation in the study. Patients with hearing difficulties or dementia, emergency procedures and procedures under general anaesthesia were excluded. Moreover, specific exclusion criteria for port procedure comprised haemorrhagic diathesis, thrombosis of jugular and subclavian veins, bilateral or acute infectious disease (e.g. sepsis or local implantation site infection) with increased risk of early port infection^18^.

### Study conduct

After patients were enrolled in the study and had given consent, they were to fill out the TAI at least 24 h prior to the procedure. Pre-interventional SAI were filled out directly prior to the intervention (< 30 min). If the participants had difficulty reading the forms, reading glasses were provided. After completing the form, patients were randomized into two study groups, the control group (CTRL) or music intervention group (MIG), using a computer-generated randomization programme (https://www.randomizer.org/). Patients allocated in the MIG were allowed to choose their desired style of music or artist. The music was played in the angio suite on a wireless stereo sound system established for that purpose exclusively (Teufel® ONE M, smart speaker). Sound volume was set to be around 50 dB.

All procedures were performed by one experienced interventionalist (F.N.F.) under peri-interventional monitoring, ensured by pulse oximetry, monitoring of blood pressure and 3-channel electrocardiogram. Following sonography and marking of the puncture site, a sterile environment was created, and local anaesthesia was administered via 1% lidocaine.

PICC access was established by the basilic or brachial vein. Both ports and CVCs were placed in the internal jugular vein and advanced to the vena cava superior. All placement procedures were performed using Seldinger-technique. Correct intravascular position of ports and CVCs was validated by fluoroscopic confirmation of an infra-diaphragmatic tip-position of the guide wire. For ports exclusively, a subcutaneous pouch approximately 3 cm below the collar bone was created after skin incision and blunt preparation down to the pectoral fascia. Consecutive steps comprised tunnelling subcutaneously from pouch to the venous puncture site on the ipsilateral lower neck, connecting the silicone catheter with the suprafascial port reservoir and suturing the system to the fascia. After flushing the catheter, correct position of the devices was radiographically confirmed and a sterile cover was applied. As part of the study, patients were asked to fill out the post-interventional SAI immediately after the procedure (< 30 min). After subjective well-being was affirmed, patients were discharged or transferred to the ward.

### Outcome parameters

Primary outcomes of this study were defined as the reduction of SAI values, meaning the difference of pre- to post-interventional stress levels, in order to analyze the influence of music exposure.

Secondary outcome parameters included mean heart rate and time of radiological surveillance for ports, which approximates the procedure duration by covering the time span from placing the guide wire to verifying the correct position of the port catheter system.

### Statistical analysis

Statistical analysis was conducted using IBM SPSS Statistics Version 26.0. Descriptive statistics were employed to examine the data and provide a broad overview. Baseline characteristics of the study population and two study groups were mapped out using means and standard deviations (SD) as primary descriptive tools. Normal distribution of the measured ordinal variables including the STAI were examined by a combination of Shapiro-Wilk-Test and visual confirmation through histograms. For normally distributed and independent variables, independent-samples t-tests with post-hoc for repeated measurement were used to compare the examined groups (MIG vs. CTRL); for normally distributed and dependent variables, paired-sample t-tests were employed. The latter applied to pre- and post-interventional SAI analysis. Categorial variables were analyzed using Chi-Square tests. Furthermore, univariate analysis of variance (ANOVA) was conducted for post-interventional SAI and the difference between pre- and post-interventional anxiety levels, adjusting for numerous variables including procedure type, familiarity with procedure, pre-interventional SAI, trait anxiety, age and sex. Of note, for the questionnaires with one or two items missing, an approximative formula was employed to enable inclusion into the analysis. Confidence intervals (CI) for statistical analysis were set at 95% and p-values of less than 0.05 were considered significant.

### Ethical approval

The study complies with the Declaration of Helsinki and was approved by the ethics committee of the Charité–Universitätsmedizin Berlin (no. EA4/079/16) on 06/29/2016. The trial was further registered with the German Clinical Trials Register (no. DRKS00026003 on 17/08/2021 and is accessible under https://www.drks.de/). All patients were informed and gave written consent to participate in the study.

## Results

**Figure 1:**
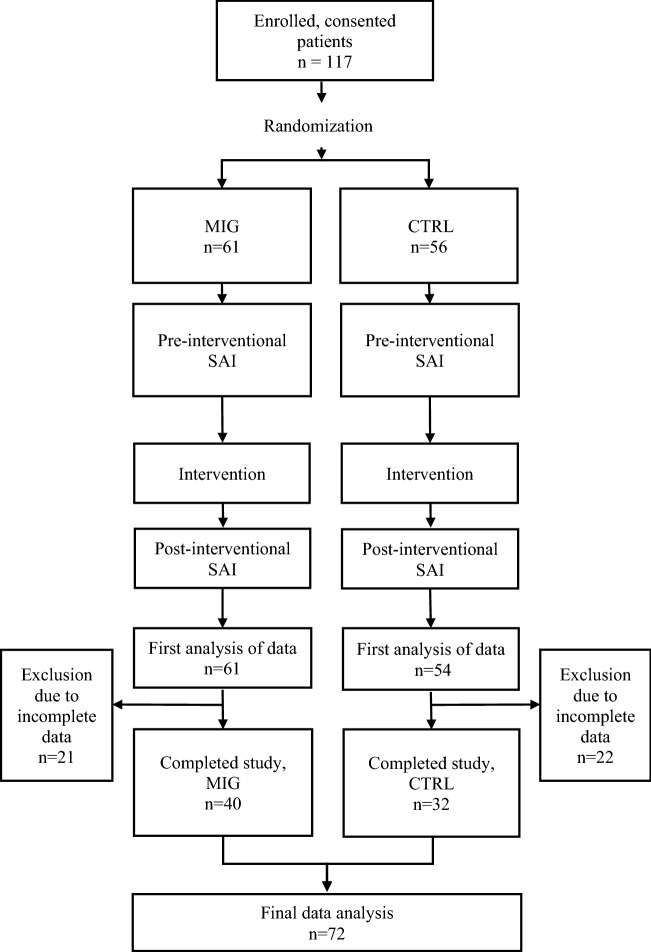
Study conduct from patient acquisition to statistical analysis. CTRL control group; MIG music intervention group; SAI state anxiety inventories.

117 patients underwent randomization and completed the procedure. No major complications were noted. 35 patients had to be excluded due to partly or completely missing questionnaires. Further 10 patients were dismissed as two or more items in the questionnaires were not properly checked. Finally, a total of 72 patients were included in the conclusive analysis (Fig. 1). Differences in the distribution of patient characteristics such as age and sex were non-significant in the MIG (*n* = 40) and the CTRL (*n* = 32). Solely, familiarity with intravenous procedures was significantly higher in the CTRL (*p* = 0.028). Baseline characteristics of the separated groups are shown in Table 1.

**Table 1:** Comparison of baseline characteristics among the two study groups.

Parameter		CTRL *n* = 32 (%)	MIG *n* = 40 (%)	Total *n* = 72	*p*-value
Sex	Male	13 (41)	25 (66)	38	0.065^b^
	Female	19 (59)	15 (44)	34	
Age		61 ± 13	59 ± 12		0.798^a^
Procedure	Port	13 (41)	19 (48)	32	0.654^b^
	PICC	12 (38)	15 (37)	27	
	CVC*	7 (21)	6 (15)	13	
Side of procedure	Left	17 (53)	20 (50)	37	0.070^b^
	Right	15 (47)	20 (50)	35	
Familiarity with procedure	Prior experience	12 (38)	6 (15)	18	*p* = 0.028^b^
	No prior experience	20 (62)	34 (85)	54	

^a^ Independent samples test (MIG vs. CTRL).

^b^ Chi-Square Test, confidence interval 95%

CTRL control group; MIG music intervention group; PICC peripherally inserted central catheter; CVC central venous catheter.

**Figure 2:**
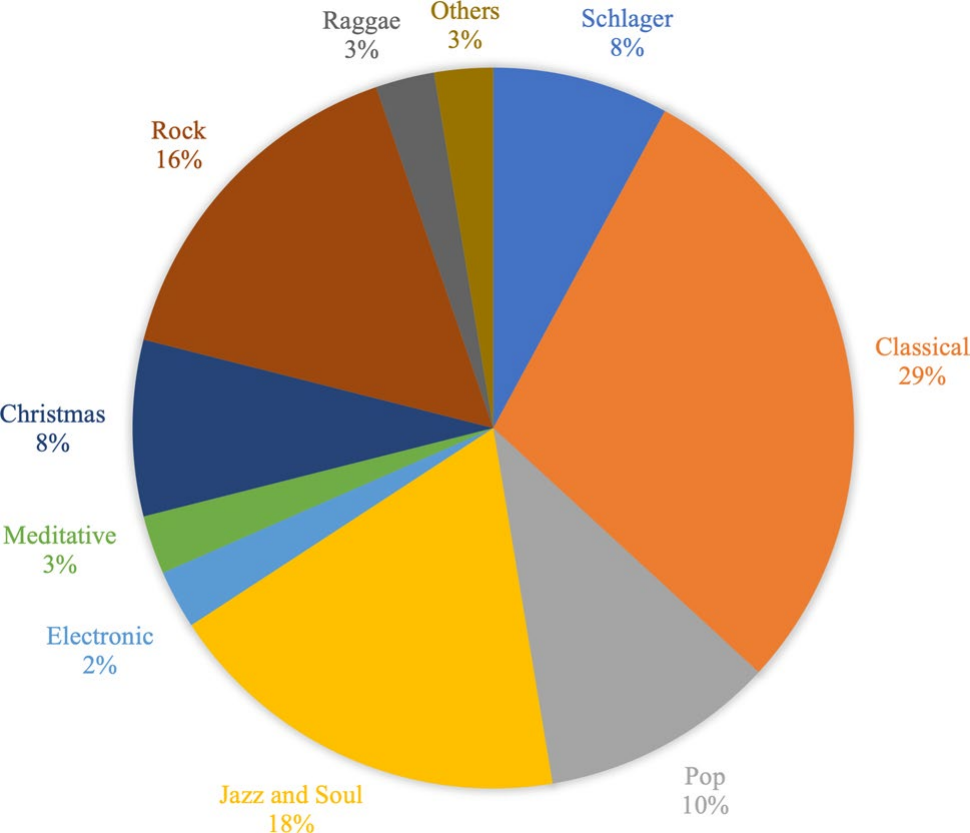
Choice of music within MIG, *n* = 40.

The choice of music ranged from German Schlager music to meditative music, with classical music being the most frequently requested (Fig. 2).

Overall, 32 patients received a port (CTRL *n* = 13, MIG *n* = 19); in 27 patients a PICC was placed (CTRL *n* = 12, MIG *n* = 15) while in 13 participants venous access was provided via CVC (CTRL *n* = 7, MIG *n* = 6). Underlying diseases of all patients according to the two study groups are shown in Table 2.

**Table 2:** Underlying diseases within CTRL and MIG.

Disease	Group allocation					
	CTRL		MIG		Total	
	*n*	%	*n*	%	*n*	%
Gastrointestinal tumors	8	25.0	8	20.0	16	22.2
Gynecological cancer	8	25.0	8	20.0	16	22.2
Lymphomatous diseases	5	15.6	6	15.0	11	15.3
Leukemia	4	12.5	4	10.0	8	11.1
Cancer of the pharynx and oral cavity	2	6.3	6	15.0	8	11.1
Lung cancer	2	6.3	4	10.0	6	8.3
Other solid tumors	1	3.1	2	5.0	3	4.2
Infectious diseases	1	3.1	0	0.0	1	1.4
Inflammatory gastrointestinal diseases	0	0.0	2	5.0	2	2.8
Not specified	1	3.1	0	0.0	1	1.4
Total	32	100.0	40	100.0	72	100.0

Pearson Chi-Square: *p* = 0.712.

CTRL control group; MIG music intervention group.

### Primary outcomes

**Figure 3:**
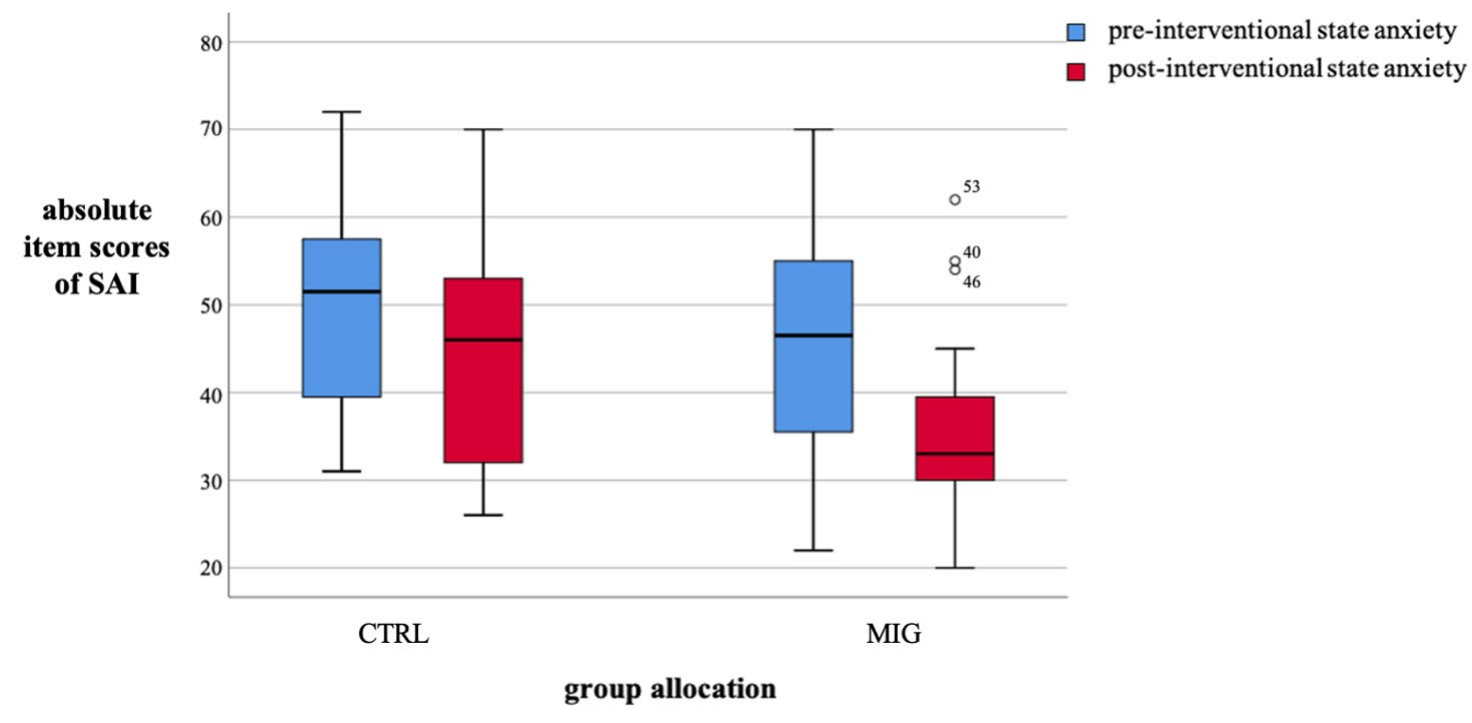
Comparison of patients’ pre- and post-interventional anxiety levels. CTRL control group; MIG music intervention group; SAI state anxiety inventories.

Mean pre- and post-interventional SAI values of both groups are shown in Table 3 and visualized in Fig. 3. Of note, initial SAI values for pre-interventional anxiety were comparable in both groups with a slightly lower mean value of 4.1 item scores in the MIG without any statistically significant difference (*p* = 0.174), visualized in Fig. 3. Post-interventional anxiety levels were lower in both groups (CTRL 50.8 ± 12.2 vs. 44.0 ± 12.1 and 46.7 ± 12.8 vs. 34.9 ± 8.9 in the MIG). The total post-interventional score for both groups ranked at 38.9 on average (SD 11.3).

**Table 3:** Patients’ pre- and post-interventional anxiety levels according to each study group.

Group allocation	Total	SAI	Mean ± SD	Mean difference pre vs. post ± SD	*p*-value
CTRL	*n* = 32	Pre	50.8 ± 12.2	6.8 ± 10.7	0.001^a^
		Post	44.0 ± 12.1		
MIG	*n* = 40	Pre	46.7 ± 12.8	11.8 ± 14.5	< 0.001^a^
		Post	34.9 ± 8.9		

^a^paired-samples t-test (pre- and post-interventional SAI).

CTRL control group; MIG music intervention group; SAI state anxiety inventories; SD standard deviation.

When compared using the unpaired-samples t-test, post-interventional anxiety scores were significantly lower in the MIG than those in the CTRL by a difference of 9.0 item scores (*p* < 0.001, Table 3).

**Figure 4:**
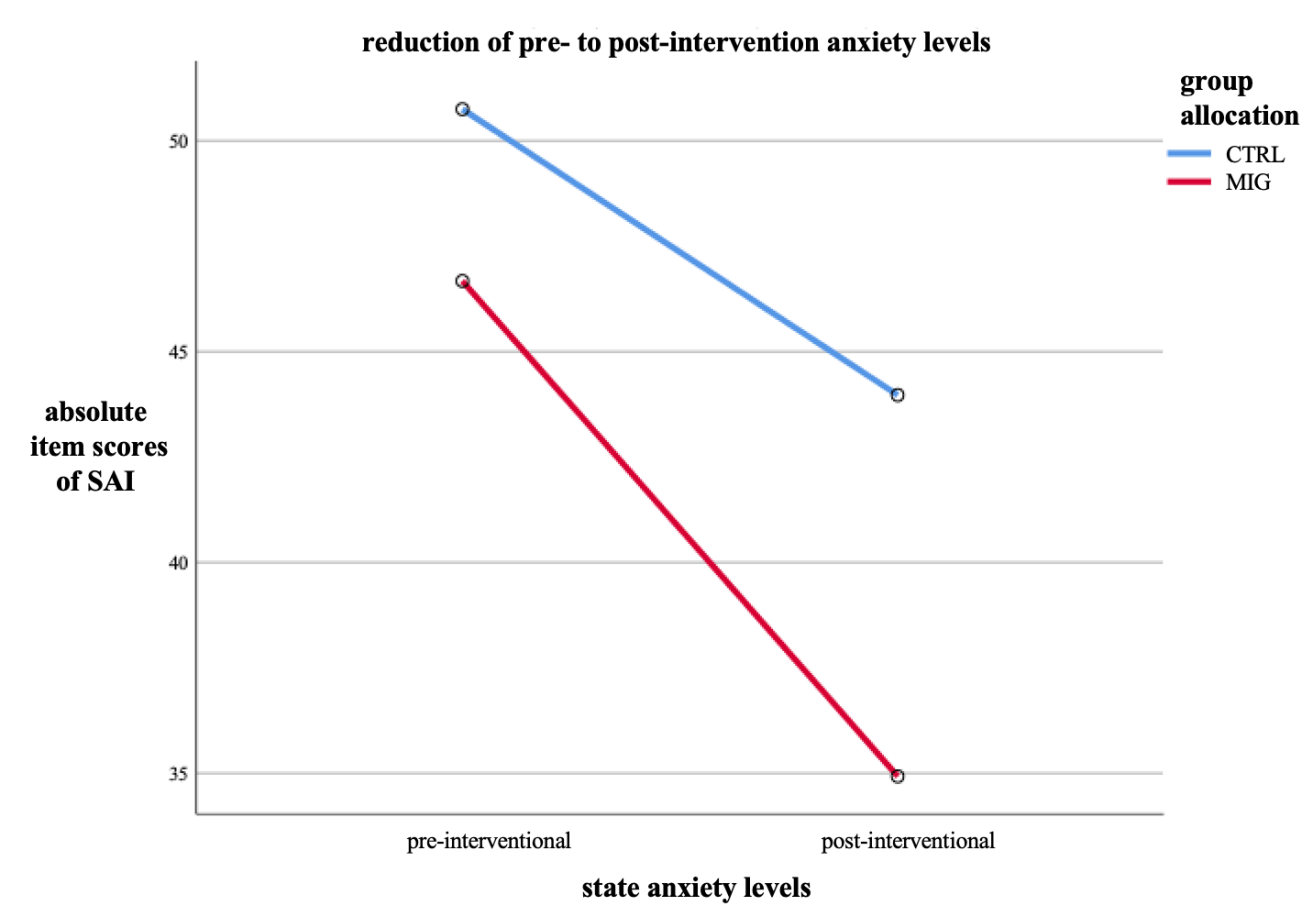
Reduction of averaged pre- and post-interventional state anxiety levels inventories among study groups as measured by the SAI, *n* = 72. CTRL control group; MIG music intervention group; SAI state anxiety inventories.

The difference in mean decline of anxiety in the MIG was 5.0 ± 3.1 item scores as compared to the CTRL (*p* = 0.110). Using the ANOVA analysis, including variables procedure type, familiarity with procedure, pre-interventional SAI, trait anxiety, age and sex, showed that the reduction of item scores was even more pronounced: music exposure reduced anxiety by − 6.9 ± 2.2 item scores (*p* < 0.001, Figs. 3 and 4).

### Secondary outcomes

As a secondary outcome, heart rate was measured during the intervention either twice or three times and consequently averaged. Measurements were done consistently in 60 patients. The mean heart rate in the MIG (*n* = 37, 76.1 ± 13.7 bpm) was significantly lower as compared to the CTRL (*n* = 23, 93 ± 8.9 bpm, *p* < 0.001).

The mean time of radiological surveillance needed during port insertion differed significantly between the study groups by approximately 3.8 min from 17.2 ± 3.6 min in the MIG (*n* = 18) to 13.4 ± 4.9 min in the CTRL (*n* = 12, *p* = 0.031). MIG ports were placed at a 5:14 ratio on the left side, whereas in the CTRL the left-to-right ratio was 3:10. A significant difference in regard to the side of port placement could be detected between both groups (*p* = 0.043).

## Discussion

The herein reported results show a marked and highly significant reduction of patients’ anxiety induced by peri-interventional music exposure in the angio suite as measured using standardized STAI questionnaires and patients’ heart rate as a vital stress parameter.

The question whether to play music in the surgical theatre has been controversially discussed in the past. Most comparable studies report a reduction of post-interventional anxiety levels largely corresponding with the results presented in this work^3,9,15,19^. Our findings show a strong effect of peri-interventional music on patients’ anxiety levels and therefore advocate for the use, based on individual needs to improve the overall experience in the angio suite. Furthermore, music clearly exerts its influence beyond the peri-procedural effects. Lower postoperative anxiety is associated with an improved recovery process, leading to shorter hospitalization time^5^. This is not only beneficial for the patient’s recovery and well-being, but also economically relevant. In comparison to alternative adjuvant anxiety therapies like meditation or hypnosis, which have been examined to achieve positive psychological and physical health outcomes, music application does not require trained staff, thereby not causing additional expenses^19^.

Music exposure can directly influence the physiological stress response which is reflected in vital parameters changes, such as heart and respiratory rate, systolic and diastolic blood pressure and hormonal blood levels^6,11^. Comparable studies reported partially opposing results regarding the music’s effectiveness in lowering those parameters^4,9,12^. In our findings, mean heart rate among music intervention recipients was significantly lower compared to the CTRL, which—as an objectifiable anxiety indicator—aligns with lower anxiety levels.

Almost all patients in our cohort suffered from oncological diseases. For these patients in particular, enhancing quality of life and reducing psychological strain is of great importance. These patients are likely to spend an extensive period of time experiencing pain and being subject to strenuous interventions in unfamiliar surroundings, very often feeling a loss of autonomy over their body and choices due to the disease^20^. In that respect and based on our results, music can be seen as a valuable component of adjuvant pain and anxiety therapy.

The perception of and reaction to music seems to be highly individual. In our data, the reduction of anxiety levels varied on a wider scale in the MIG. This finding might indicate that the effects on each participant may have differed dependant on their general attitude towards and emotional connection to the music chosen. This must be taken into account, especially since the soothing effects of music are partially dependent on emotional components. Yet, in contrast to all positive effects, some studies find music to be a potential source of distraction by hampering communication in the operation theatre^5,21,22^. This hypothesis however, is highly controversial since other publications found music not to obstruct physician–patient-communication, even making everyone calmer and in that way more efficient^19,21^. In that respect, music exposure was reported to achieve higher speed and accuracy in task performance by enhancing concentration and executive skills^7,21^. Our data showed significantly longer duration times for port placement procedures by 3:45 min in the MIG (*p* = 0.031). An explanation for this might be that the number of port procedures on the left side was significantly higher in the MIG (*p* = 0.043), whereby longer duration may be attributed to more complex anatomical conditions^18^. Nonetheless, the question whether music might lower the interventionalist’s performance cannot be answered by this study design. Of note, further investigation on objectifiable effects of music on staff and the quality of procedures itself is underway.

This study has several limitations. Firstly, the study was performed at a single, yet maximum-care centre. However, all procedures were conducted by the same experienced interventional radiologist creating equal conditions and thus improving internal validity. Secondly, due to the per-protocol approach of this study only a part of all patients was enrolled into the final analysis. Thirdly, due to expected lower post-operative STAI values there is a possible bias from repeated measures. Lastly, as for the nature of music, blinding is impossible. This might have caused bias, especially the Hawthorne effect.

## Conclusion

Music can act as a safe, easily applicable, free of charge and non-invasive aid to reduce anxiety in the peri-operative setting to improve quality of care. Apart from statistical significances, we believe our findings are of high clinical relevance beyond the field of IR.
